# The *Pseudomonas syringae* pv. tomato DC3000 effector HopD1 interferes with cellular dynamics associated with the function of the plant immune protein AtNHR2B

**DOI:** 10.3389/fmicb.2023.1305899

**Published:** 2023-11-23

**Authors:** Luis Francisco Marín-Ponce, Catalina Rodríguez-Puerto, Perla Rocha-Loyola, Clemencia M. Rojas

**Affiliations:** ^1^Cell and Molecular Biology Program, University of Arkansas, Fayetteville, AR, United States; ^2^Department of Entomology and Plant Pathology, University of Arkansas, Fayetteville, AR, United States

**Keywords:** *Pseudomonas syringae* pv. tomato DC3000, type III secreted effectors, HopD1, AtNHR2B, cellular dynamics, vesicle trafficking

## Abstract

The plant pathogenic bacterium *Pseudomonas syringae* pv tomato DC3000 (*Pst* DC3000) causes disease in tomato, in the model plant *Arabidopsis thaliana,* and conditionally in *Nicotiana benthamiana.* The pathogenicity of *Pst* DC3000 is mostly due to bacterial virulence proteins, known as effectors, that are translocated into the plant cytoplasm through the type III secretion system (T3SS). Bacterial type III secreted effectors (T3SEs) target plants physiological processes and suppress defense responses to enable and support bacterial proliferation. The *Pst* DC3000 T3SE HopD1 interferes with plant defense responses by targeting the transcription factor NTL9. This work shows that HopD1 also targets the immune protein AtNHR2B (*Arabidopsis thaliana* nonhost resistance 2B), a protein that localizes to dynamic vesicles of the plant endomembrane system. Live-cell imaging of *Nicotiana benthamiana* plants transiently co-expressing *HopD1* fused to the epitope haemagglutinin (*HopD1-HA*) with *AtNHR2B* fused to the red fluorescent protein (*AtNHR2B-RFP*), revealed that HopD1-HA interferes with the abundance and cellular dynamics of AtNHR2B-RFP-containing vesicles. The results from this study shed light into an additional function of HopD1 while contributing to understanding how T3SEs also target vesicle trafficking-mediated processes in plants.

## 1 Introduction

The bacterium *Pseudomonas syringae* is a ubiquitous and diverse bacterium species complex that can be isolated from a wide range of habitats, including agricultural environments (Morris et al., 2013). *P. syringae* strains isolated from agricultural habitats have been intensely investigated as they cause diseases in a wide range of plant species (Mansfield et al., 2012). Among the pathogenic strains of *P. syringae*, *Pseudomonas syringae* pv. tomato DC3000 has acquired particular importance for its ability to cause disease in the model plant *Arabidopsis thaliana* and conditionally on *Nicotiana benthamiana* (Wei et al., 2007; Xin and He, 2013).

All the plant pathogenic strains of *P. syringae* possess a *hrp/hrc* gene cluster that encodes a type III secretion system (T3SS), a complex of proteins assembled in the inner and outer bacterial membranes; the T3SS extends outwards with an extracellular filament termed the Hrp pilus, through which bacterial proteins, known as type III secreted effectors (T3SEs), are translocated directly into the plant cytoplasm (Alfano and Collmer, 2004). Translocation of T3SEs results in two opposite outcomes depending on the specific *P. syringae* strain and plant combination. In nonhost plants, T3SE are recognized directly or indirectly by plants nucleotide-binding leucine-rich repeat receptors (NLRs), triggering Effector-Trigger Immunity (ETI) that culminates in a process of programmed cell death called the hypersensitive response (HR). In host plants, T3SEs promote bacterial parasitism by interfering with plant defense responses, altering essential biological functions and promoting pathogen proliferation, ultimately resulting in disease development (Wei et al., 2018).

The complexity, diversity and abundance of T3SEs has been uncovered by analyzing the genomic sequence of 494 *P. syringae* strains (Dillon et al., 2019). Interestingly, while each *P. syringae* strain has a unique number and repertoire of effectors, four core effectors: AvrE, HopB1, HopM1 and HopAA1, were identified in more than 95% of the strains, highlighting their importance in basic interactions with plants, or in pathogenesis (Bundalovic-Torma et al., 2022). The function of the remaining effectors in each strain is difficult to assess for the following reasons: (i) a given effector can have several targets, (ii) multiple effectors are redundant, having the same function, or targeting the same plant component, and (iii) effectors can interact with other effectors in multiple combinations. The generation of *Pst* DC3000 polymutants with sequential deletion of its well-expressed 28 effectors and further combinatorial reassembly of effectors has significantly contributed to untangle the complexity of *Pst* DC3000 by evaluating their growth in *N. benthamiana* (Kvitko et al., 2009; Cunnac et al., 2011). With that approach, eight effectors were identified as the minimal repertoire required for pathogenesis of *Pst* DC3000 in *N. benthamiana*: AvrPtoB, HopM1, HopE1, HopG1, HopAM1-1, AvrE1, HopAA1-1 and HopN1 (Kvitko et al., 2009; Cunnac et al., 2011). In addition to those effectors, HopD1 was also identified as an important effector to enable *Pst* DC3000 growth in *N. benthamiana* and *Arabidopsis* (Kvitko et al., 2009; Block et al., 2014), likely due to its ability to suppress ETI (Block et al., 2014). Moreover, transgenic plants expressing *HopD1* enhanced the growth of the avirulent *Pst* DC3000 strains: *Pst* DC3000 (*avrRpm1*) and *Pst* DC3000 (*avrRpt2*), which in non-transgenic plants are detected by the plant immune system preventing their proliferation (Block et al., 2014).

Previously, AtNHR2B was identified as a protein participating in plant immunity, as the *Atnhr2b* mutant was susceptible to the non-adapted pathogen *P. syringae* pv. tabaci, which is unable to cause disease and proliferate in wild-type Arabidopsis plants (Singh et al., 2018). Moreover, AtNHR2B fused to the green fluorescent protein (AtNHR2B-GFP) localizes to cytoplasm, chloroplasts and small dynamic vesicles of unknown identity, which represent compartments of the endomembrane system that participate in vesicle trafficking-mediated events such as endocytosis and secretion (Singh et al., 2018). Interestingly, inoculation of Arabidopsis *AtNHR2B-GFP*-transgenic lines with the adapted pathogen *Pseudomonas syringae* pv tomato DC3000 (*Pst* DC3000), which is capable of causing disease in Arabidopsis, alters the localization and abundance of AtNHR2B-GFP (Rodriguez-Puerto et al., 2022). This finding led us to hypothesize that *Pst* DC3000 type III secreted effectors (T3SE) acting as virulence proteins target AtNHR2B. Given the importance of HopD1 in *Pst* DC3000 virulence (Kvitko et al., 2009; Block et al., 2014), we decided to investigate whether AtNHR2B could be an additional target for HopD1. The results showed that HopD1 physically interacts and partially co-localizes with AtNHR2B in *N. benthamiana*, and that HopD1 reduces the abundance and cellular dynamics of AtNHR2B-containing vesicles. These results provide direct evidence that *Pst* DC3000 hijacks the plant immune system by interfering with vesicle trafficking events.

## 2 Materials and methods

### 2.1 Plant materials and growth conditions

*N. benthamiana* seeds were sown on potting soil (PRO mix LP15), kept at 4°C for 2 days in the dark to break dormancy and transferred to a growth chamber set up at 21°C with a photoperiod of 8 h of light and 16 h of darkness. Seeds were grown for 2 weeks, and single seedlings were transplanted to individual pots and grown for two more weeks.

### 2.2 Plasmids

Full length *AtNHR2B* in the entry vector *pDONR201* was recombined into vector *pK7RWG2* to generate *AtNHR2B-RFP, pK7FWG2::HopD1-GFP* and *pPZ212::HopD1-HA* (Block et al., 2014), were obtained from Dr. Jim Alfano, University of Nebraska-Lincoln.

### 2.3 Bacterial strains

Plasmid *pK7RWG2::AtNHR2B, pK7FWG2::HopD1-GFP* and *pPZ212::HopD1-HA* were transformed into *Agrobacterium tumefaciens* GV2260 and plated on Luria Bertani (LB) supplemented with rifampicin (50 μg/mL) and streptomycin (50 μg/mL). Glycerol stocks were generated from individual transformants obtained.

### 2.4 Yeast-two hybrid assay

*AtNHR2B* and *HopD1* were cloned into bait and prey vectors of the ProQuest™ Two-Hybrid System (Invitrogen, Carlsbad, CA). Full length *AtNHR2B* in the entry vector *pDONR201* was recombined into the bait vector *pDEST22* to generate a fusion to the *GAL4* transcriptional activation domain. *HopD1* fused to the *GAL4* DNA binding domain in *pDEST32* vector was synthesized by Gene Universal (Newark, DE).

Yeast strain MaV203 competent cells (Thermo Fisher Scientific, Waltham, MA) were transformed with yeast two-hybrid constructs using the Frozen-EZ Yeast Transformation II Kit (Zymo Research, Irvine, CA). The following combinations of constructs were co-transformed into yeast: *pDEST22* + *pDEST32*::*HopD1* and *pDEST22*::*AtNHR2B* + *pDEST32::HopD1*. The combination of constructs (*pEXP22*™ */RalGDS-*wt + *pEXP32*™*/Krev1*) and (*pEXP22*™ */RalGDS-*m1 + *pEXP32*™*/Krev1*), provided by the manufacturer (Thermo Fisher Scientific, Waltham, MA), were used as positive and negative controls, respectively. Transformed yeast cells were plated on Double Drop Out (DDO) selection plates lacking amino acids leucine and tryptophan and grown at 30°C for 4 days. Single colonies were picked from the plates and cultured in 15 mL DDO broth at 30°C overnight. The overnight culture was diluted to an OD_600_nm of 0.2 and plated on Triple Drop Out (TDO) selection plates lacking amino acids leucine, tryptophan and histidine and supplemented with 15 mM 3-Amino-1,2,4-Triazole (3-AT) and grown at 30°C for 4 days.

### 2.5 Transient expression in *Nicotiana benthamiana*

*Agrobacterium tumefaciens* strains harboring constructs of interest, depending on the experiment, were streaked out on LB plates containing rifampicin (50 μg/mL) and streptomycin (50 μg/mL); single colonies were transferred to LB broth with respective antibiotics, and grown with constant aeration for 18 h. Cultures were harvested by centrifugation at 6,000 rpm for 10 min and supernatants were discarded. Bacterial pellets were resuspended in 5 mL induction buffer (20 mM MES pH 5.5; Mannitol; 200 mM acetosyringone) and incubated for 4 h at room temperature with slow agitation. After induction, bacteria were harvested by centrifugation at 6,000 rpm for 10 min, and pellets resuspended in 5 mL resuspension buffer (10 mM MES pH 5.5). Bacterial concentration was adjusted to a final concentration of OD_600_ of 0.3 and infiltrated into fully expanded leaves of four-week-old *N. benthamiana* plants.

### 2.6 Co-immunoprecipitation

*A. tumefaciens* harboring *HopD1-HA* was co-infiltrated either with *A. tumefaciens* strains harboring *AtNHR2B-GFP* or *A. tumefaciens* harboring free *GFP* into fully expanded leaves of four-week-old *N. benthamiana* plants. Infiltrated leaves were collected at 3 days post-infiltration and tissue was ground in liquid nitrogen for protein extraction. Tissue powder was homogenized in 5 mL of co-immunoprecipitation extraction buffer (100 mM Tris–HCl, pH 7.5, 150 mM NaCl, 1 mM EDTA, 10 mM MgCl2, 10% Glycerol, 0.2% Nonidet P-40, 1 mM PMSF, 5 mM DTT, 1X Proteinase inhibitor cocktail) (Sigma Aldrich, St. Louis, MO) and incubated on ice for 30 min. Samples were centrifuged at 4°C for 30 min at 13,000 rpm and supernatants containing extracted proteins were collected in a pre-chilled 50 mL falcon tube.

Before co-immunoprecipitation, protein concentration was measured by Bradford Assay (BioRad, Hercules, CA) and proteins of interest were confirmed by running protein samples into a SDS-PAGE gel followed by Western blot with Anti-GFP-HRP (1:1000 dilution; Miltenyi Biotec, Auburn, CA) and Anti-HA-HRP (1:1000 dilution; Thermo Fisher Scientific Inc., Carlsbad, CA) and detected by luminol solution (ImmunoCruz, SantaCruz Biotechnology Inc., Dallas, TX).

For co-immunoprecipitation, 1 mg of total protein extract was mixed with 20 μL of PierceTM HA Epitope Tag Antibody conjugated to agarose beads (Thermo Fisher Scientific, Waltham, MA) and incubated overnight at 4°C with end-to-end rocking. After incubation, protein complexes bound to beads were washed three times with 1X TBS buffer (50 mM Tris–HCl, 150 mM NaCl, pH 7.5). Protein complexes bound to the beads were eluted in 2× SDS protein loading buffer, loaded and ran into an SDS-PAGE gel and transferred to nitrocellulose membranes. Proteins were detected by Western Blot with anti-GFP-HRP or anti-HA-HRP antibodies.

### 2.7 Imaging for co-localization

*N. benthamiana* plants transiently co-expressing *AtNHR2B-RFP* and *HopD1-GFP* were imaged at 2 days post-inoculation (dpi) using the Leica Stellaris 8 laser scanning confocal microscope at 20× magnification. Images were acquired in sequential scanning mode in the GFP (excitation wavelength: 496 nm, emission wavelength: 549 nm) and RFP (excitation wavelength: 570 nm, emission wavelength: 657 nm) channels.

Five independent images were chosen for colocalization analysis. Images in microscope-specific format (.lif) files were imported into FIJI, converted into TIFF files and pre-processed to remove background noise and to adjust brightness and contrast using automatic function. Green and read channels were split into separate images, and the overlapped image was selected for analysis. BIOP’s (Bioimaging and optics Platform) JACoP (Just Another Colocalization Plugin) plugin was used to evaluate co-localization of AtNHR2B-RFP and HopD1-GFP and to calculate Pearson’s Correlation coefficient.

### 2.8 Imaging to evaluate AtNHR2B-RFP dynamics

*N. benthamiana* plants transiently expressing *AtNHR2B-RFP* alone or *AtNHR2B-RFP* in combination with *HopD1-HA* were imaged at 2 dpi using the Leica Stellaris 8 laser scanning confocal microscope. Images were acquired using the XYT image acquisition mode, to generate 4 videos of 120 frames for each treatment. Each video was taken using identical parameters: 1024 × 512, 400 bps, with line averages of 1, line accuracies of 1, frame averages of 1, and frame accuracies of 1.

Tracking of AtNHR2B-RFP-containing vesicles in each treatment was done with the software IMARIS. Video files in microscope-specific format (.lif) were converted into IMARIS-specific format (.ims) files which were used for subsequent data processing and analysis. Using the Spot Objects option and the circle selection mode, vesicles of 0.75 μm in diameter were chosen for analysis. A total of 400 vesicles for each treatment (*AtNHR2B-RFP* or *AtNHR2B-RFP/HopD1-HA*) were tracked in automated mode for speed and displacement using software’s default parameters.

Data obtained from speed and displacement was imported into R version 4.2.1. and sorted into 30 bins of equal range. Speed was sorted into bins ranging from 0.00738 μm/s to 2.56 μm/s and displacement was sorted into bins ranging from 0.00518 to 13.93 μm.

## 3 Results

### 3.1 HopD1 interacts with AtNHR2B in yeast and *in planta*

**Figure 1:**
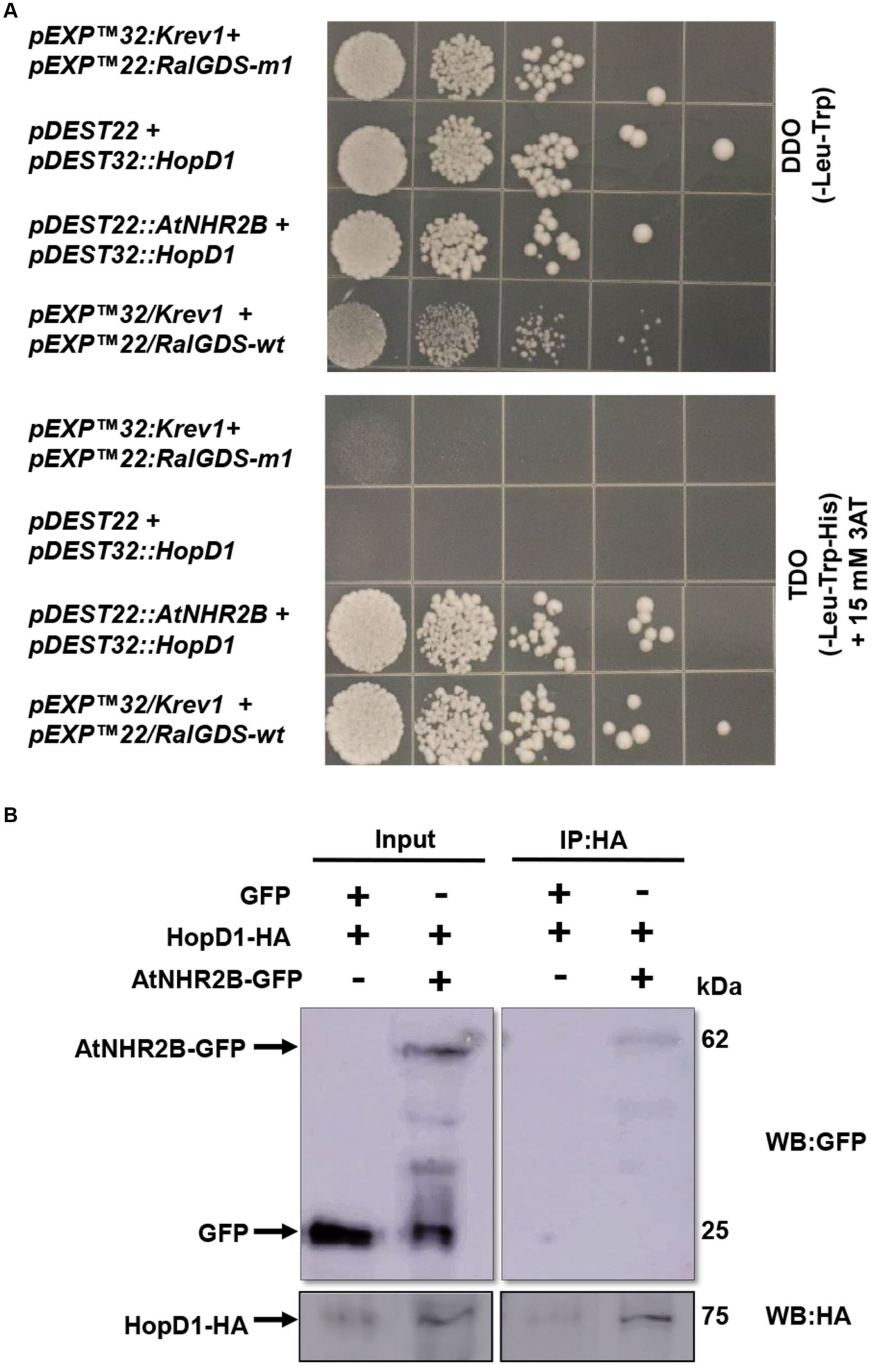
HopD1 interacts AtNHR2B in yeast and *in planta*. Yeast two-hybrid system was used to evaluate the interaction between HopD1 and AtNHR2B. Yeast strain MaV203 was co-transformed with yeast two-hybrid constructs and plated on double drop out (DDO – Leucine-Tryptophan) (top) or triple drop out (TDO – Leucine-Tryptophan-Histidine) and supplemented with 3-Amino-1,2,4-Triazole (3-AT) (bottom) **(A)**. *Agrobacterium tumefaciens* strains harboring *HopD1-HA* and *AtNHR2B-GFP* were co-infiltrated into four-weeks *N. benthamiana* plants for transient expression. As control *Agrobacterium tumefaciens* harboring *HopD1-HA* was co-infiltrated with *Agrobacterium tumefaciens* harboring a construct expressing *35S-GFP*. Infiltrated leaves were harvested for protein extraction followed by immunoprecipitation using anti-HA antibodies. Immunoprecipitated samples were separated by SDS-PAGE electrophoresis and transferred to a nitrocellulose membrane for Western Blot analysis using anti-HA and anti-GFP antibodies **(B)**.

The previous findings that HopD1 in is important in *Pst* DC3000 virulence (Kvitko et al., 2009; Block et al., 2014), led us to hypothesize that HopD1 targets AtNHR2B. To start testing this hypothesis, we initially evaluated the physical interaction between both proteins in yeast given its simplicity. The interaction in yeast was evaluated by co-transforming *AtNHR2B* and *HopD1* into yeast. The results show that all the co-transformations grew in DDO, lacking leucine and tryptophan, demonstrating the presence of both bait and prey vectors. Plating on the highly selective media TDO lacking leucine, tryptophan and histidine and supplemented with 15 mM 3 AT, showed growth of yeast strains co-expressing AtNHR2B and HopD1, demonstrating that HopD1 interacts with AtNHR2B in yeast. Yeast growth on TDO + 3AT was also observed with the positive controls, as expected. The observed growth when yeast was co-transformed with AtNHR2B and HopD1 represents a true interaction, and not the result of auto-activation as yeast strains co-transformed with HopD1 and the empty vector *pDEST22* were not able to grow in the highly selective media (Figure 1A).

To validate the yeast interaction *in planta,* we transiently co-expressed *HopD1-HA* with either *AtNHR2B-GFP* or *GFP* in *N. benthamiana.* Infiltrated leaves were collected for protein extraction followed by immunoprecipitation with anti-HA antibodies. The results show that HA antibodies immunoprecipitated HopD1-HA as expected, and co-immunoprecipitated AtNHR2B-GFP. HA antibodies did not co-immunoprecipitate free GFP, demonstrating that HopD1 physically interacts with AtNHR2B and not with the GFP tag (Figure 1B).

### 3.2 HopD1-GFP partially co-localizes with AtNHR2B-RFP

**Figure 2:**
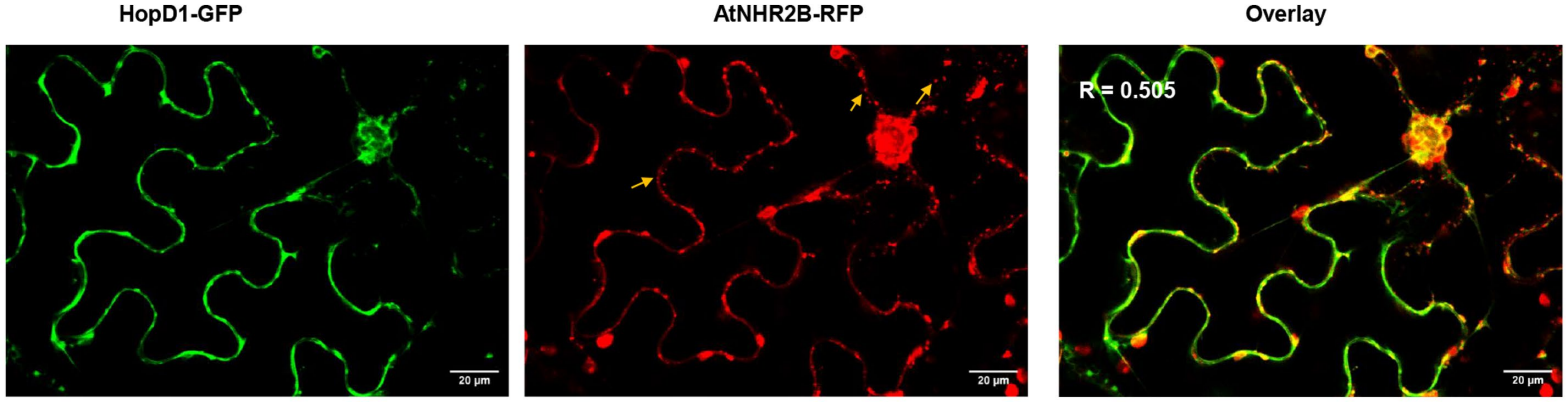
HopD1 partially colocalizes with AtNHR2B. Four-week-old *Nicotiana benthamiana* plants were co-infiltrated with two *A. tumefaciens* strains harboring *AtNHR2B-RFP* and *HopD1-GFP*. At 2dpi, plants were imaged on a Leica Stellaris 8 laser scanning confocal microscope at 20 X magnification by sequential scanning using GFP and RFP channels. Yellow areas in the overlay reveal areas of co-localization. Yellow arrows show AtNHR2B-RFP-containing vesicles. Pearson’s coefficient analysis (R) was calculated by Image J.

The physical interaction between HopD1 and AtNHR2B in yeast and *in planta*, prompted our interest to start dissecting how HopD1 targets AtNHR2B. For that purpose, we evaluated the co-localization *in situ* between HopD1 and AtNHR2B by transiently co-expressing *HopD1-GFP* with *AtNHR2B-RFP* in *N. benthamiana* and monitoring the co-localization by live-cell imaging. The results showed that AtNHR2B-RFP localizes to cytoplasm and small vesicles as previously described (Singh et al., 2018), whereas HopD1-GFP predominantly localizes to cytoplasm, and occasionally to small vesicles (Figure 2). Interestingly, overlaying the images obtained from the green and red channels revealed co-localization areas (yellow signal) mostly in small vesicles (Figure 2, Supplementary Figure S1). The co-localization of HopD1 and AtNHR2B is not complete and Pearson’s correlation coefficient analysis calculated for 5 images ranged between 0.361 and 0.633 implying a positive but partial co-localization (Figure 2, Supplementary Figure S1). The differences in the Pearson’s correlation coefficients might be related to the dynamic nature of AtNHR2B-RFP transitioning through subcellular compartments.

### 3.3 HopD1 reduces the abundance of AtNHR2B-containing vesicles

**Figure 3:**
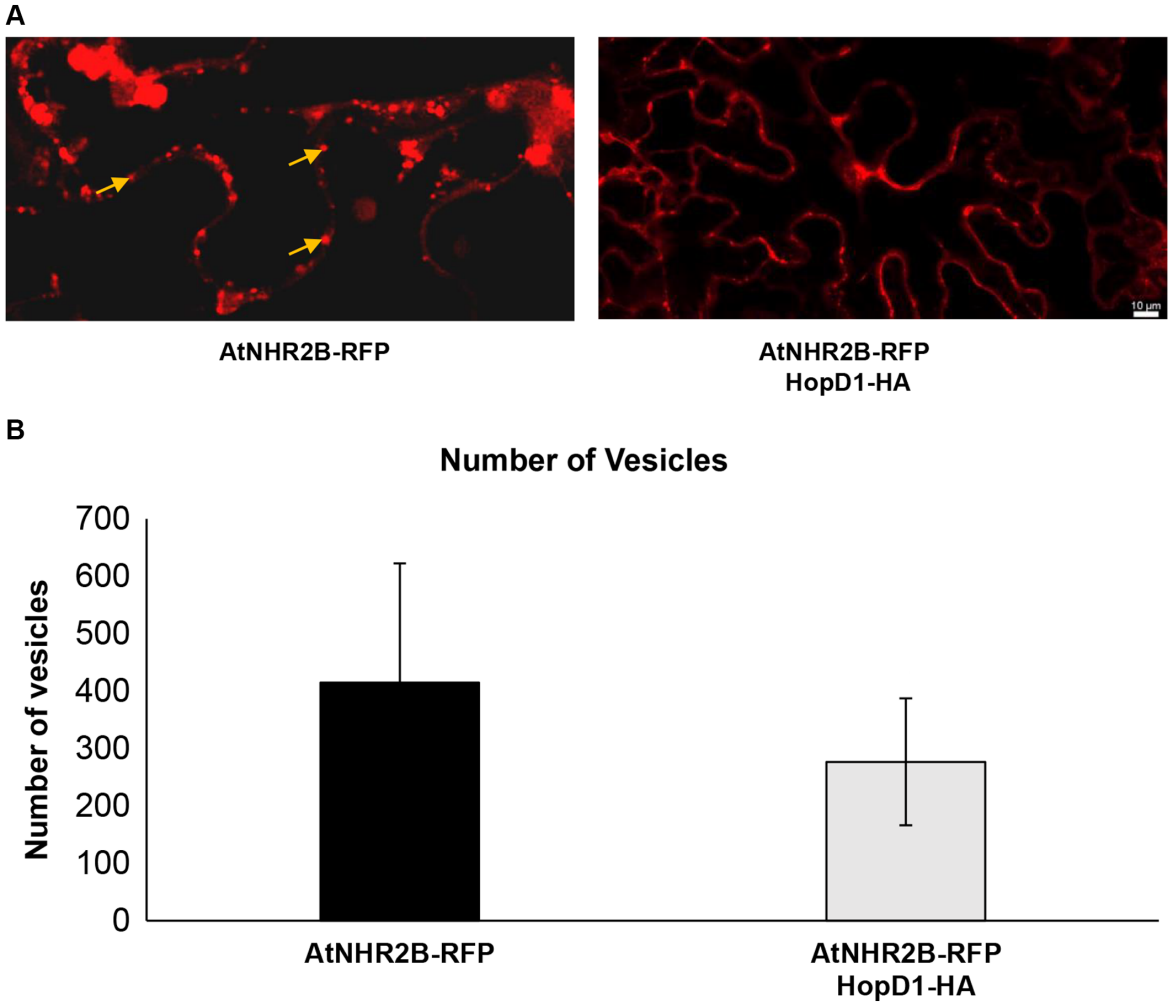
HopD1 interferes with the abundance of AtNHR2B-containing vesicles. Four-week-old *Nicothiana benthamiana* plants were infiltrated with *A. tumefaciens* harboring *AtNHR2B-RFP* or co-infiltrated with *two A. tumefaciens* strains harboring *AtNHR2B-RFP and HopD1-HA*. Plants were imaged at 2 days post-infiltration using a Leica Stellaris 8 laser scanning confocal microscope at 20× magnification **(A)**. Images collected from 120 independent frames from three experiments were used to quantify the number of vesicles using software Imaris. Bars indicate average number of vesicles per treatment. **(B)**. Arrows in **(A)** show AtNHR2B-RFP-containing vesicles. No statistically significant difference according to Mann–Whitney test were observed in the number of vesicles in **(B)**.

The finding that HopD1 co-localizes with AtNHR2B, together with our previous findings that AtNHR2B localizes to highly dynamic vesicles, led us to hypothesize that HopD1 interferes with cellular processes mediated by AtNHR2B. To test this hypothesis, we evaluated the effect of HopD1 on the dynamics of AtNHR2B-RFP-containing vesicles by live-cell imaging in *N. benthamiana.* For that purpose, *AtNHR2B-RFP* alone or in combination with *HopD1-HA* were transiently expressed in *N. benthamiana.* Live-cell imaging of *N. benthamiana* plants transiently expressing *AtNHR2B-RFP* alone confirmed its localization to cytoplasm and to small, highly dynamic vesicles, as reported previously (Singh et al., 2018). In contrast, *N. benthamiana* plants transiently co-expressing *AtNHR2B-RFP* with *HopD1-HA,* showed a decrease in the fluorescent signal and a dramatic reduction in the overall number of AtNHR2B-RFP-containing vesicles (Figure 3A). To confirm these results, time-lapse images of *N. benthamiana* plants expressing *AtNHR2B-RFP* alone, and plants co-expressing *AtNHR2B-RFP* and *HopD1-HA* were taken to quantify the number of AtNHR2B-RFP-containing vesicles in plants expressing *AtNHR2B-RFP* alone in comparison with plants co-expressing *AtNHR2B-RFP* with *HopD1-HA.* The results from 120 frames for 4 independent videos for each of the treatments, showed that plants expressing *AtNHR2-RFP* alone had on average number of 415 AtNHR2B-RFP-containing vesicles, whereas plants co-expressing *AtNHR2B-RFP* with *HopD1-HA* had on average of 277 (Figure 3B). Unfortunately, although the videos revealed differences between both experimental conditions, the difference in quantification was not statistically significant, likely due to the large margin of error and the relatively small sample size.

### 3.4 HopD1 interferes with AtNHR2B speed

Time-lapse images of AtNHR2B-RFP-containing vesicles showed the dynamic movement patterns of AtNHR2B-RFP-containing vesicles (Supplementary Video S1). Those dynamic patterns of movement of AtNHR2B-RFP-containing vesicles are significantly altered and reduced in the presence of HopD1 (Supplementary Video S2). To evaluate this phenomenon in more detail, AtNHR2B-RFP-containing vesicles were tracked to quantify their speed under two experimental conditions: in *N. benthamiana* plants expressing *AtNHR2B-RFP* alone, and in *N. benthamiana* co-expressing *AtNHR2B-RFP* and *HopD1-HA.* To make meaningful comparisons and to prevent biased results arising from the differences in the number of AtNHR2B-RFP-containing vesicles between experimental conditions, the same number of vesicles were evaluated for each of the 4 videos per experimental condition, tracking a total of 400 vesicles for each experimental condition.

**Figure 4:**
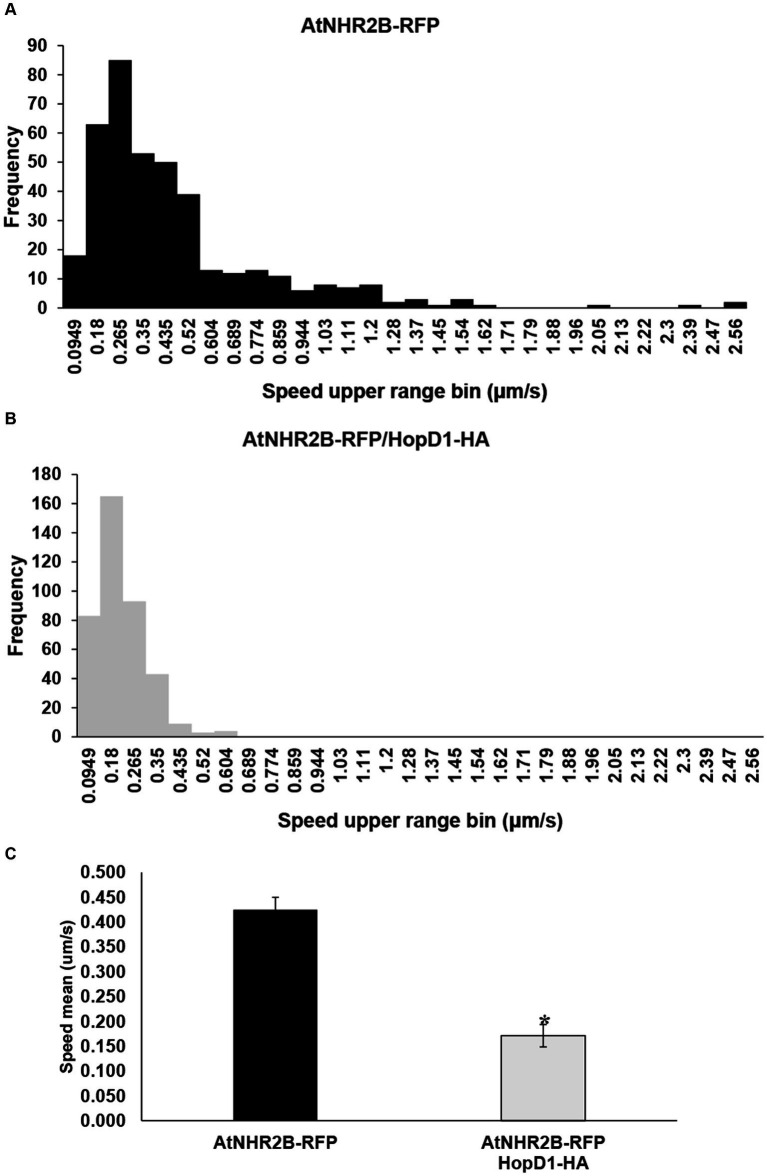
The type III effector HopD1 interferes with the speed of AtNHR2B-containing vesicles. Four-week-old *N. benthamiana* plants were infiltrated with with *A. tumefaciens* harboring *AtNHR2B-RFP* or co-infiltrated with two *A. tumefaciens* strains harboring *AtNHR2B-RFP* and *HopD1-HA*. Infiltrated leaves were imaged at 2 dpi, and four videos of 120 frames were captured for each treatment using a Leica Stellaris 8 laser scanning confocal microscope. Videos were analyzed with Imaris Microscopy Image Analysis software to sort out the AtNHR2B-RFP-containing vesicles into different populations (bins) based on speed and with or without HopD1-HA **(A,B)**, and to calculate the average speed with and without HopD1-HA **(C)**. Bars in **(C)** represent average and standard deviation for each of the measurements for each treatment. Asterisk in **(C)** indicates statistically significant difference by Student’s *t*-test.

Sorting the AtNHR2B-RFP-containing vesicles by speed into 30 bins ranging from 0.00738 to 2.56 μm/s revealed that AtNHR2B-RFP*-*containing vesicles from *N. benthamiana* plants expressing *AtNHR2B-RFP* alone span 22 of the 30 bins, and their abundance in each of those bins ranged from 1 to 85. More than 10 vesicles per bin were observed in bins 1–10, that cumulatively cover speeds from 0.00738 to 0.859 μm/s (Table 1). The largest number of AtNHR2B-RFP-containing vesicles (85) underwent selective sorting into the bin corresponding to speeds between 0.18 μm/s and 0.265 μm/s (Table 1). In contrast to AtNHR2B-RFP-containing vesicles from *N. benthamiana* plants expressing *AtNHR2B-RFP* alone, *N. benthamiana* plants co-expressing *AtNHR2B-RFP* and *HopD1-HA* were sorted into 7 of the 30 different bins spanning a range of 0.00738 to 0.604 μm/s. In this experimental condition, only bins 1–4 have more than 10 vesicles, and the bin containing the largest number of AtNHR2B-RFP-containing vesicles was observed in the bin corresponding to speeds between 0.0949 μm/s and 0.18 μm/s (Table 1). Altogether the data shows that expression of *AtNHR2B-RFP* alone generate a diverse population of AtNHR2B-RFP-containing vesicles with a wider range of speeds, include fast moving vesicles (Figure 4A), whereas the co-expression of *AtNHR2B-RFP* with *HopD1-HA* generates fewer types of AtNHR2B-RFP-containing vesicles sorted into slow speeds (Figure 4B) and slowest total mean speed. In addition, the mean speed of AtNHR2B-GFP-containing vesicles in *N. benthamiana* plants expressing *AtNHR2B-RFP* alone was 0.425 μm/s, whereas the mean speed for AtNHR2B-RFP-containing vesicles in *N. benthamiana* plants co-expressing *AtNHR2B-RFP* and *HopD1-HA* was 0.171 μm/s (Figure 4C). These findings demonstrate that HopD1 significantly interferes with the speed of AtNHR2B-RFP-containing vesicles.

**Table 1:** Effect of HopD1-HA on the speed of AtNHR2B-RFP-containing vesicles

		Number of vesicles per experimental condition	
Bins	Speed range	AtNHR2B-RFP	AtNHR2B-RFP/HopD1-HA
1	0.00738–0.0949	18	83
2	0.0949–0.18	63	165
3	0.18–0.265	85	93
4	0.265–0.35	53	43
5	0.35–0.435	50	9
6	0.435–0.52	39	3
7	0.52–0.604	13	4
8	0.604–0.689	12	0
9	0.689–0.774	13	0
10	0.774–0.859	11	0
11	0.859–0.944	6	0
12	0.944–1.03	8	0
13	1.03–1.11	7	0
14	1.11–1.2	8	0
15	1.2–1.28	2	0
16	1.28–1.37	3	0
17	1.37–1.45	1	0
18	1.45–1.54	3	0
19	1.54–1.62	1	0
20	1.62–1.71	0	0
21	1.71–1.79	0	0
22	1.79–1.88	0	0
23	1.88–1.96	0	0
24	1.96–2.05	1	0
25	2.05–2.13	0	0
26	2.13–2.22	0	0
27	2.22–2.3	0	0
28	2.3–2.39	1	0
29	2.39–2.47	0	0
30	2.47–2.56	2	0

### 3.5 HopD1 interfere with AtNHR2B displacement

**Figure 5:**
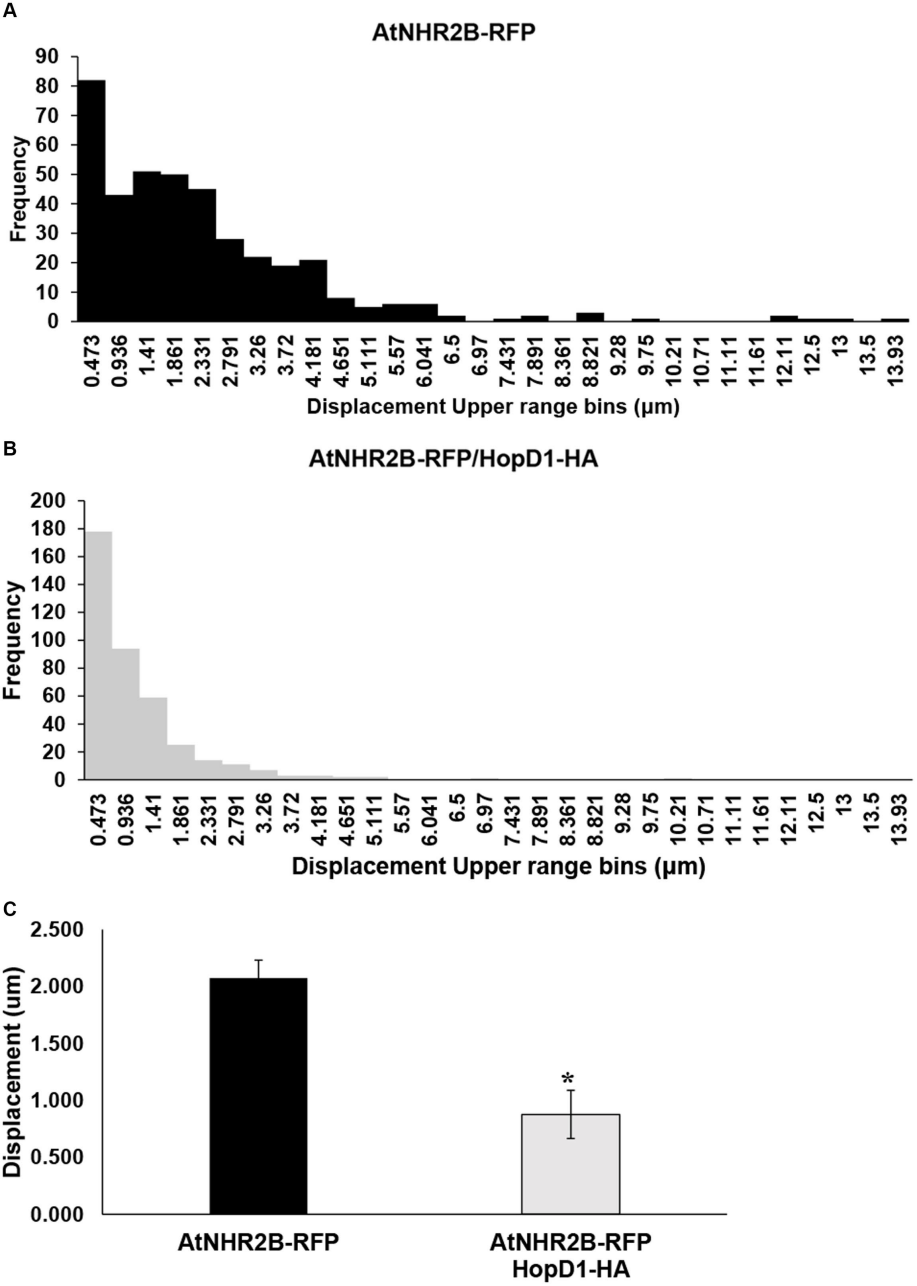
The type III effector HopD1 interferes with the displacement of AtNHR2B-containing vesicles. Four-week-old *N. benthamiana* plants were infiltrated with *A. tumefaciens* harboring *AtNHR2B-RFP* or co-infiltrated with two *A. tumefaciens* strains harboring *AtNHR2B-RFP* and *HopD1-HA*. Infiltrated leaves were imaged at 2 dpi, and four videos of 120 frames were captured for each treatment using a Leica Stellaris 8 laser scanning confocal microscope. Videos were analyzed with Imaris Microscopy Image Analysis software to sort out the AtNHR2B-RFP-containing vesicles into different populations (bins) based on displacement and with or without HopD1-HA **(A,B)**, and to calculate the average speed with and without HopD1-HA **(C)**. Bars in **(C)**. represent average and standard deviation for each of the measurements for each treatment. Asterisk in **(B)** indicates statistically significant difference by Student’s *t*-test.

To further evaluate the effect of HopD1 on the dynamics of AtNHR2B-RFP-containing vesicles, the same time-lapse images were used to evaluate displacement of AtNHR2B-RFP-containing vesicles. AtNHR2B-RFP-containing vesicles from plants expressing *AtNHR2B-RFP* alone and from plants co-expressing *AtNHR2B-RFP* and *HopD1-HA* were sorted into 30 bins ranging from 0.00518 μm to 13.93 μm. The results revealed that plants expressing *AtNHR2B-RFP* alone were sorted into 22 of the 30 bins and their abundance in each bin ranges from 1 to 82. Bins 1–9 contain more than 10 vesicles; the largest number of AtNHR2B-RFP-containing vesicles were observed in bin 1 corresponding to displacement ranging from 0.00518 μm to 0.473 μm (Table 2). The displacement of AtNHR2B-RFP-containing vesicles in *N. benthamiana* plants co-expressing *AtNHR2B-RFP* and *HopD1-HA* were sorted into only 13 of the 30 available and their abundance in each bin ranged from 1 to 178. More than 10 vesicles/bin were observed in bins 1–6 and the largest number of vesicles (178) were found bin 1 corresponding to displacements between 0.00518 μm and 0.473 μm (Table 2). When comparing the bins in common for both experimental conditions (*AtNHR2B-RFP* alone versus *AtNHR2B-RFP/HopD1-HA*), the results show a similar pattern of distribution to the pattern observed in speed, wherein AtNHR2B-RFP-containing vesicles obtained from plants expressing *AtNHR2B-RFP* alone showed a wider range of displacement but less abundance (Figure 5A), whereas AtNHR2B-RFP-containing vesicles obtained from plants co-expressing *AtNHR2B-RFP* with *HopD1-HA,* were aggregated into a narrower range of bins, corresponding to lower displacement, and with larger numbers of vesicles per bin (Figure 5B). Moreover, the mean displacement of AtNHR2B-RFP-containing vesicles was 2.072 μm in *N. benthamiana* plants expressing *AtNHR2B-RFP* alone, whereas the mean displacement for AtNHR2B-RFP-containing vesicles in *N. benthamiana* plants co-expressing *AtNHR2B-RFP* and *HopD1-HA* was 0.876 μm. Altogether, these findings demonstrate that HopD1 actively reduces the displacement of AtNHR2B-RFP-containing vesicles (Figure 5C).

**Table 2:** Effect of HopD1 on the displacement of AtNHR2B-RFP containing vesicles.

		Number of vesicles per experimental condition	
Bins	Displacement range	AtNHR2B-RFP	AtNHR2B-RFP/HopD1-HA
1	0.00518–0.473	82	178
2	0.473–0.936	43	94
3	0.936–1.41	51	59
4	1.4–1.861	50	25
5	1.86–2.331	45	14
6	2.33–2.791	28	11
7	2.79–3.26	22	7
8	3.26–3.72	19	3
9	3.72–4.181	21	3
10	4.18–4.651	8	2
11	4.65–5.111	5	2
12	5.11–5.57	6	0
13	5.57–6.041	6	0
14	6.04–6.5	2	0
15	6.5–6.97	0	1
16	6.97–7.431	1	0
17	7.43–7.891	2	0
18	7.89–8.361	0	0
19	8.36–8.821	3	0
20	8.82–9.28	0	0
21	9.28–9.75	1	0
22	9.75–10.21	0	1
23	10.2–10.71	0	0
24	10.7–11.11	0	0
25	11.1–11.61	0	0
26	11.6–12.11	2	0
27	12.1–12.5	1	0
28	12.5–13	1	0
29	13–13.5	0	0
30	13.5–13.93	1	0

## 4 Discussion

Bacterial pathogens such as *Pst* DC3000 employ T3SE to interfere with two main branches of plant immunity: Pathogen-Associated Molecular Patterns (PAMP)-triggered immunity (PTI) and Effector-Triggered Immunity (ETI) (Jones and Dangl, 2006). PTI is initiated when plants recognize common features present in the surface of microbes/pathogens and known as Microbe-Associated Molecular Patterns (MAMPs)/ Pathogen-Associated Molecular Patterns (PAMPs) (Zhang and Zhou, 2010). PAMP recognition is achieved by Pathogen Recognition Receptors (PRRs) located on the plant surface (Saijo et al., 2018), and subsequent signal transduction requires the phosphorylation of PRRs and interaction with co-receptors such as BAK1 (Couto and Zipfel, 2016; Yu et al., 2017; Saijo et al., 2018). To counteract PTI, *Pst* DC3000 T3SE redundantly target PRRs as well as proteins that participate in PTI signaling. For example, T3SE AvrPto and AvrPtoB target PRRs such as FLS2, EFR and CERK1 (Xiang et al., 2008; Gimenez-Ibanez et al., 2009), and prevent the binding of flg22 to FLS2 (Shan et al., 2008), HopAO1 with tyrosine phosphatase activity prevents phosphorylation of EFR (Underwood et al., 2007; Macho et al., 2014), and HopB1 targets BAK1 (Li et al., 2016). T3SE also redundantly suppress ETI (Jamir et al., 2004; Guo et al., 2009), and one of those effectors is HopD1 (Block et al., 2014).

HopD1 localizes to the endoplasmic reticulum (ER) where it interacts with the ER-localized transcription factor NTL9, which regulates the expression of ETI-related genes (Block et al., 2014). This work showed that HopD1 also interacts with the immune-related protein AtNHR2B. The co-localization of HopD1-GFP with AtNHR2B-RFP was found to be in cytoplasm and in small subcellular compartments (vesicles) of the endomembrane system where AtNHR2B was previously found to be localized (Singh et al., 2018). The localization of AtNHR2B to compartments of the endomembrane systems suggests a function in vesicle trafficking which mediates cellular processes such as endocytosis and secretion (Jeon and Segonzac, 2023). During plant immunity, endocytosis mediates the internalization of PAMPs-activated PRR which in turn induces downstream signaling pathways involved in plant defense (Robatzek et al., 2006; Chinchilla et al., 2007; Ben Khaled et al., 2015; Mbengue et al., 2016; Gu et al., 2017).Vesicle trafficking events also mediate secretory processes, which in the context of plant immunity are responsible for the remodeling of the cell wall, the release of antimicrobials to the apoplast and the replenishing of PRR to the plasma membrane (Kwon et al., 2008; Kim and Brandizzi, 2014).

While it is still not known if AtNHR2B participates in endocytosis or secretion, the results from this work clearly demonstrate that HopD1 alters the speed and displacement of AtNHR2B-RFP carrying vesicles. Targeting endocytosis and secretion by T3SE is not unexpected given their importance in plant immunity (Gu et al., 2017), and examples of effectors targeting those processes are starting to emerge. For example, HopM1 localizes to endomembrane compartments, specifically to trans-Golgi network/early endosomes (Nomura et al., 2011), and targets AtMIN7 (*Arabidopsis thaliana* HopM1 interactor 7), a vesicle trafficking component required for callose deposition (Nomura et al., 2006). AvrPtoB targets EXO70B1 a subunit of the exocyst complex which plays a vital role in the tethering of secretory vesicles to the plasma membrane for subsequent exocytosis (Wang et al., 2019). HopG1 and HopW1 bind to actin and disrupt actin cytoskeleton and endocytosis interfering with vesicle trafficking (Jelenska et al., 2014; Kang et al., 2014; Shimono et al., 2016), whereas HopZ1a, an acetyltransferase destroys microtubule networks interfering with secretion (Lee et al., 2012). HopE1 also interferes with the microtubule network by interacting with MAP65 (microtubule-associated protein 65) causing its dissociation from the microtubule network and affecting secretion as well (Guo et al., 2016). The results from this work provide compelling evidence that HopD1 interferes with vesicle trafficking processes mediated by AtNHR2B but the specific processes affected still remain to be characterized.

## Data availability statement

The original contributions presented in the study are included in the article/Supplementary material, further inquiries can be directed to the corresponding author.

## Author contributions

LM-P: Formal analysis, Methodology, Writing – original draft, Writing – review & editing. CR-P: Investigation, Methodology, Writing – review & editing. PR-L: Investigation, Methodology, Writing – review & editing. CR: Conceptualization, Funding acquisition, Investigation, Resources, Supervision, Visualization, Writing – original draft, Writing – review & editing.
