# Supplementary figures and images for: The Pseudomonas syringae pv. tomato DC3000 effector HopD1 interferes with cellular dynamics associated with the function of the plant immune protein AtNHR2B
Source: Front Microbiol. 2023 Nov 23;14:1305899. doi: 10.3389/fmicb.2023.1305899 (PMC10702356; doi:10.3389/fmicb.2023.1305899)

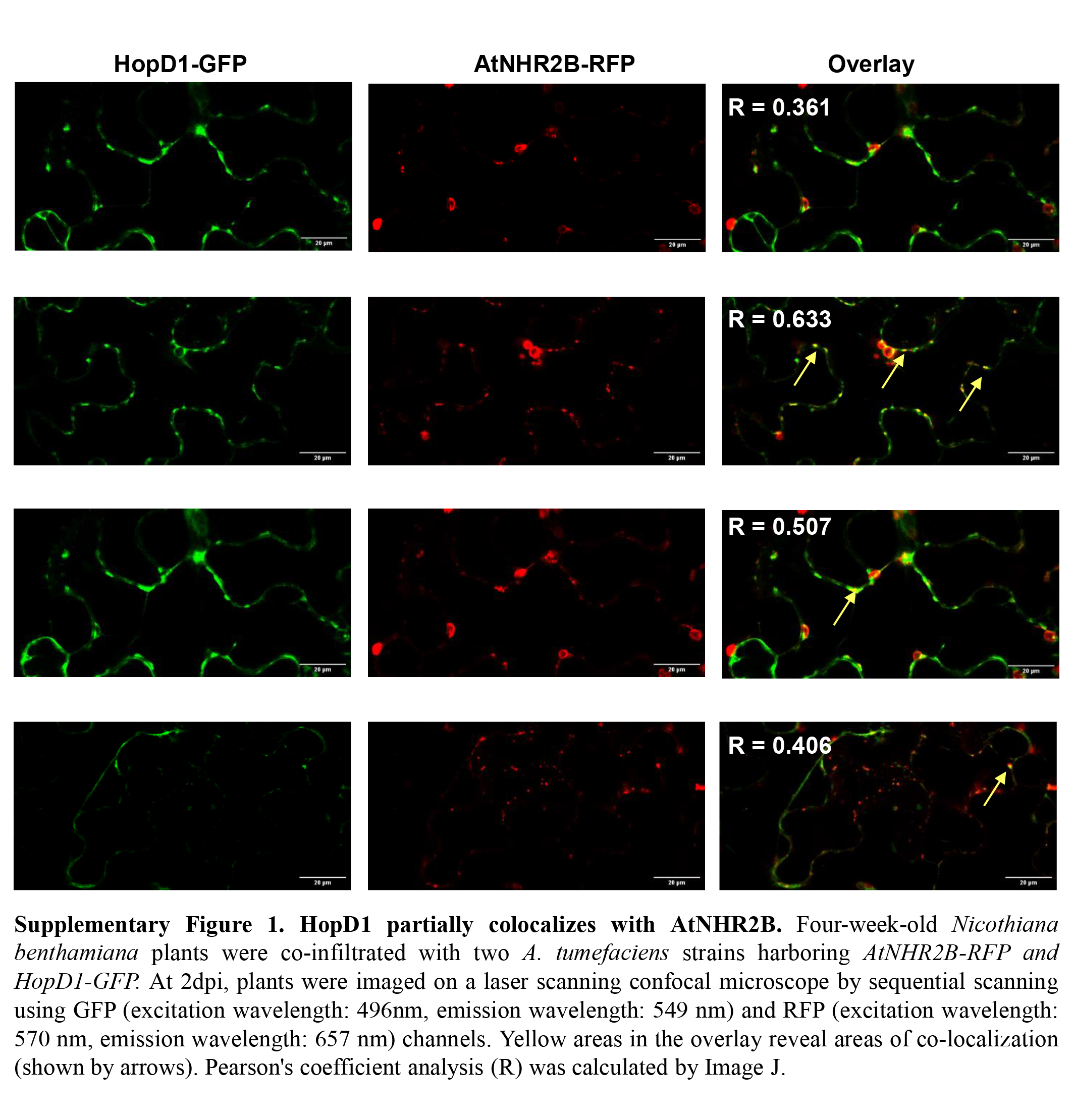

Supplement: Supplementary file 1 [file Image_1.TIF]
